# Prescriptions of homeopathic remedies at the expense of the German statutory health insurance from 1985 to 2021: scientific, legal and pharmacoeconomic analysis

**DOI:** 10.1007/s00210-024-03005-x

**Published:** 2024-03-02

**Authors:** Hauke Leemhuis, Roland Seifert

**Affiliations:** Institute of Pharmacology, Hannover Medical School, Carl-Neuberg-Straße 1, D-30625 Hannover, Germany

**Keywords:** Homeopathy, Evidence levels, DDD, SHI system, Rational pharmacological alternative, Pubmed.gov

## Abstract

The prescription of homeopathic remedies at the expense of the statutory health insurance (SHI) system in Germany has been criticized for years due to a lack of evidence. Now, on the planned abolition of the reimbursement of homeopathic medicines in Germany, the debate on this topic has been reignited. The aim of this paper is to show the costs and their development over time incurred by homeopathic remedies in the healthcare system from 1985 to 2021. For this purpose, 15 selected homeopathic medicines were chosen from the drug prescription report (Arzneiverordnungsreport) and analyzed with regard to their development of DDD (Defined Daily Dose) using data from the Wissenschaftliches Institut der Ortskrankenkassen (WidO, Scientific Institute of the General Local Health Insurance Funds) and compared with their respective rational pharmacological alternatives. The price comparison was based on the DDD costs and the pharmacy retail price of the smallest packaging in each case. The clinical study situation for the preparations was also analyzed. For this purpose, the clinical studies provided by the manufacturer and those on PubMed were divided into evidence levels and analyzed. In addition, the presentation of homeopathic remedies on company websites, in online pharmacies, in specialist information and package leaflets was analyzed with regard to side effects, interactions, indication, and information on the alleged effect/proof of efficacy. In many media, information on homeopathic medicines remained incomplete, and non-compliance with the Therapeutic Product Advertising Act (Heilmittelwerbegesetz) was noted. Naming of the products if often very suggestive, too. Manufacturers’ claims of efficacy go far beyond what can be considered proven in terms of evidence-based medicine and the quality of most clinical studies is poor. Homeopathic remedies are on average significantly more expensive than their rational pharmacological alternatives. Furthermore, DDD costs have continued to rise over the years analyzed. In aggregate, from a pharmacoeconomic, legal, and scientific perspective, abolition of reimbursement of homeopathic medicines in Germany at the expense of the SHI system is well justified.

## Introduction

On 11 January, 2024, Germany’s Federal Health Minister Prof. Dr. Karl Lauterbach stated that services that do not have a proven medical benefit may not be financed by the SHI. The study quality of homeopathic studies and the approval procedures for these drugs are a matter of current debate (Siggelkow and Leimbach 2024). The reimbursement of homeopathic remedies should be critically analyzed not only for economic reasons, but also due to the external impact of the financing of homeopathic manufacturers, who position themselves contrary to evidence-based medicine.

Homeopathy is a type of complementary and alternative medicine (National Health and Medical Research Council 2015) that dates back to ideas and principles of Samuel Hahnemann in the late eighteenth century (Stub et al. 2022). Even though homeopathy originates from Germany, it is used around the world with over 200 million people using homeopathy on a regular basis worldwide (Commission of the European Communities 1997, Prasad 2007). In the European Union, three out of four people know about homeopathy and of these three about 29% use homeopathy for their health care (Commission of the European Communities 1997). Homeopathy is practiced in 40 out of 42 countries in Europe and included in the national health systems of many countries worldwide, such as Brazil, Chile, India, Mexico, Pakistan, and Switzerland (World Health Organization 2001). Over 6 million people use homeopathy in the USA. Of these 1 million are children and over 5 million are adults (Black et al. 2015; Clarke et al. 2015).

Homeopathy is based on the simile principle (like cures like). Diseases are treated with substances that would cause similar symptoms in a healthy person (National Health and Medical Research Council 2015). According to homeopathic teaching, however, the decisive factor is potentization, in which the starting substance is diluted and shaken in a certain way. According to homeopathic understanding, the effect becomes stronger the more often this step is repeated, even if, from a scientific point of view, it is merely a matter of dilution, which fundamentally contradicts the dose–response principle (Mukerji and Ernst 2022). For example, a substance that causes an increase of body temperature in healthy people could be used as a medicament against fever after homeopathic potentization. However, many homeopathic remedies are diluted to such an extent that they do not contain a single molecule of the starting substance (Mukerji and Ernst 2022). Homeopaths have often tried to prove the effect of homeopathic remedies in clinical studies, but numerous large meta-analyses have shown that there is no reliable evidence for the effect of homeopathy beyond the placebo effect (Antonelli and Donelli 2019, Cucherat et al. 2000, Kleijnen et al. 1991, Linde and Melchart 1998, Linde et al. 1997, Mathie et al. 2014, Mathie et al. 2017, Mathie et al. 2018, Mathie et al. 2019, National Health and Medical Research Council 2015, Shang et al. 2005). For these reasons, there has long been a critical debate in Germany as to whether homeopathy should continue to be financed with SHI funds. At the heart of the debate is the question of whether the funding of treatments that contradict the principles of evidence-based medicine (Lübbers and Endruscheit 2021) can still be justified.

In 2021, the SHI system spent € 22 million on homeopathic and anthroposophical remedies (Siggelkow and Leimbach 2024). According to the federal association of the pharmaceutical industry (Bundesverband der Pharmazeutischen Industrie), sales of homeopathic remedies stabilized in 2022 with sales of 44.7 million packagings after the market for homeopathic remedies experienced a slight decline during the coronavirus pandemic, as did the entire OTC (over-the-counter) market. In Germany, homeopathic remedies generated sales of around € 607 million in 2022. Self-medication accounted for the lion’s share at € 534 million. Around € 74 million was attributable to prescriptions for these medicines. These prescriptions can be submitted to statutory health insurers and partially reimbursed. (Bundesverband der Pharmazeutischen Industrie e., V. (BPI) 2023).

Since the adoption of the GKV-Modernisierungsgesetz (SHI Modernization Act) 2003, the prescription of homeopathic remedies is no longer part of the SHI benefits catalogue. However, they can still be reimbursed by the statutory health insurance scheme via statutory benefits. Statutory benefits are voluntary additional benefits provided by the respective health insurance funds, with which the health insurance funds compete with each other (Bundesministerium für Gesundheit 2016). To provide this discussion with scientific data, we critically analyzed the market of homeopathic medicines in Germany using stringent pharmacoeconomic and pharmacological criteria.

## Methods

In the following analysis based on data from the Scientific Institute of the General Local Health Insurance Fund (WIdO) (https://www.wido.de/, as of 16 August 2023), the DDD, DDD costs, and total sales of 16 homeopathic medicines from 1985 to 2021 were examined. The preparations were selected on the basis of the AVR, which presents the 3000 leading drugs on the German pharmaceutical market. It covers all homeopathic remedies listed in the AVR from 2005 to 2022 (Ludwig et al. 2021, 2022; Schwabe and Paffrath 2006, 2007, 2008a, 2008b, 2009, 2010, 2011, 2012, 2013, 2014, 2015, 2016; Schwabe et al. 2017, 2018, 2019; Schwabe and Ludwig 2020). Anthroposophic and phytotherapeutic medicines were not included. The WIdO data refer to medicines prescribed by registered doctors in outpatient care and dispensed via public pharmacies at the expense of the SHI system. In addition, only medicines that were regularly prescribed via the “SHI prescription” were recorded. Private prescriptions which most probably provide a large share of the market could unfortunately not be recorded due to lack of data available to us. The WIdO data were then used to analyze the course of the individual parameters over time. Of these 16 homeopathic remedies, those for which the WIdO was able to provide consistent data from 1985 to 2021 were then identified. These were the following: HR2, HR5, HR11, HR12, HR13, HR14, and HR16 (Table 1).

**Table 1:** Investigated homeopathic remedies with composition, indication, and rational pharmacological alternative. One of the underlying principles producing homeopathic remedies is potentiation. Therefore, the starting substance is either diluted in a ratio of 1:10 (D) or 1:100 (C). The number behind the letter indicates how often this step is repeated with the resulting dilution. For example, D6 equals a dilution of 1:1,000,000 (Klein 2023)

Code of homeopathic remedy (HR)	Composition	Indication	Rational pharmacological alternative
HR1	Aconitum D5 Atropinum sulf. D5 Mercurius cyanatus D8	Cold remedy	Ibuprofen, Paracetamol, Metamizole
HR2	Arnika D3 Calendula Ø Hamamelis Ø Echinacea ang. Ø Echinacea purp. Ø Chamomilla Ø Symphytum D4 Bellis perennis Ø Hypericum D6 Millefolium Ø Aconitum D1 Belladonna D1 Mercurius sol. D6 Hepar sulfuris D6	Diseases of the musculoskeletal system	Diclofenac, Ibuprofen
HR3	Euphorbium D4 Pulsatilla D2 Mercurius biiod. D8 Hepar sulfuris D10 Argentum nitr. D10 Luffa operculate D2	Diseases of the upper respiratory tract	Ibuprofen, Paracetamol
HR4	Chamomilla D1 Belladonna D2 Plantago major D3 Pulsatilla D2 Calcium carbonicum Hahnemanni D8	Restlessness in infants and toddlers	Paracetamol suppositories, Ibuprofen
HR5	Aconitum D6 Capsicum D4 Chamomilla Ø Echinacea purp. Ø Hydrastis D4 Hydrargyrum D6 Jodum D4 Natrium tetraboracicum D4 Sambucus nigra Ø Sanguinaria Ø	Otitis media	Xylometazoline, Ibuprofen, Paracetamol, Metamizole
HR6	Echin. Angustifolia Ø Aconitum Ø Belladonna Ø Eupatorium Perfol. Ø	Feverish, flu-like infections with inflammation of the upper airways	Ibuprofen, Paracetamol, Metamizole
HR7	Carum Carvi	(intestinal colic)	Butylscopolamine
HR8	Capsicum annum D3 Guaiacum D3 Phytolacca americana ø	Acute inflammation of the throat and tonsils (tonsillitis)	Ibuprofen, Paracetamol, Metamizole
HR9	Cinnabaris D8 Carbo vegetabilis D8 Silicea D8 Mercur. solub. D8 Kalium bichromic. D4 Calc. sulfuric. D4 Hydrastis D4 Thuja D8	Sinusitis	Xylometazoline, Ibuprofen, Paracetamol, Metamizole
HR10	Apis D4 Baptisia D4 Cinnabaris D3 Echinacea D2 Hepar sulfuris D3 Kalium bichromic. D8 Lachesis D8 Luffa D4 Mercurius bijodatus D9 Silicea D2 Spongia D6	Sinsusitis	Xylometazoline, Ibuprofen, Paracetamol, Metamizole
HR11	Magnesium phosph. C6 Calcium carb. „Hahnemanni “ C8 Chamomilla D6 Calcium phosph. D12 Ferrum phosporicum C8	Teething problems	Ibuprofen, Paracetamol
HR12	Sonnentau Ø Hedera helix Ø China D1 Cochenillelaus D1 Kupfersulfat D1 Ipecacuanha D4 Hyoscyamos D4	Irritable cough	Codeine, Noscapine
HR13	Atropin. sulf. D5 Hepar sulf. D3 Kalium bichrom. D4 Silicea D2 Merc. biiodat. D8	Inflammation of the throat	Ibuprofen, Paracetamol, Metamizole
HR14	Colocynthis D4 Ammonium brom. D4 Atropinum sulf. D6 Veratrum D6 Magnesium phosph. D6 Gelsenium D6 Passioflora Inc. D2 Chamomilla D3 Cuprum sulf. D6 Amanita muscaria D4 Aconitum D6	Cramp-like complaints with digestive disorders	Butylscopolamine
HR15	Chamomilla D12 Kal. phosphoric. D6 Staphisagria D12 Valeriana D6	Nervous disorders with restlessness	Methylphenidate (only for attention deficit hyperactivity disorder (ADHD) diagnosed by qualified psychiatrists)
HR16	Aconitum D4 Bryonia D4 Lachesis D12 Eupatorium D3 Phosphor D5	Flu-like infections	Ibuprofen, Paracetamol, Metamizole

In a second step, rational pharmacological alternatives were then identified and compared with the homeopathic medicines using the DDD costs from 2021. In addition, the pharmacy retail price of the smallest pack in each case was analyzed in comparison. For the homeopathic remedies, the prices of the smallest pack of each application and dosage form were included in the analysis.

### Investigated homeopathic remedies

The condition for inclusion of a preparation was that it had been listed in the AVR at least once between 2005 and 2022. Anthroposophic and phytotherapeutic remedies were not included. Homeopathic remedies were coded by numbers (HR1-HR16). The specific product names are available from the authors upon reasonable request. The analyzed remedies are listed in Table 1.

With one exception (HR7 suppositories), the preparations analyzed were homeopathic complex preparations, i.e., preparations containing several individual homeopathic remedies. The number of “active ingredients” per preparation ranges from one to 14 (HR2). The potencies of the active ingredients range from D1 to C8 (HR11). This means that these are exclusively preparations with relatively low potency levels, where it can generally be assumed that the “active ingredient” is not diluted to such an extent that it can no longer be detected (Kammler 2021). An effect would therefore be conceivable in principle, but should be proven in clinical studies, as with any medication.

Of the homeopathic remedies analyzed, 14 were authorized remedies and two were only registered. This is because most homeopathic complex remedies are not eligible for registration as they use potencies like D3 and below (see section “Registration”).

### Determination of the rational pharmacological alternative

The respective indication was determined for each preparation based on the package leaflet. For registered homeopathic medicinal products, one “indication” was analyzed as an example (see Table 1). Active substances were then sought for these preparations that correspond to a rational pharmacological therapy. The cheapest preparation from the AVR 2021 was then selected for these active substances (example: HR15 N→ Indication: Nervous disorders with restlessness→ Rational pharmacological alternative: Methylphenidate→ cheapest preparation in the AVR 2021: Methylphenidat AL). The following drugs were analyzed as rational pharmacological alternatives:

OTC: Ibuprofen, Paracetamol, Xylometazoline, Butylscopolamine, Diclofenac.

Prescription: Metamizole, Codeine, Noscapine, Methylphenidate.

To match the application forms of the corresponding homeopathic remedy, for some substances, several preparations were analyzed.

### Examination of the clinical evidence of efficacy

In a further step, representative clinical studies that can be found for the respective preparations on PubMed (https://pubmed.ncbi.nlm.nih.gov/, as of 16 August 2023) and those clinical studies that the manufacturers state on their company websites were then analyzed with regard to their evidence class (Table 2). The findings of these analyses were then compared with the information provided by the manufacturers on their websites regarding efficacy/proof of efficacy. It should be noted that this refers to the evidence class that the study was intended to achieve, not necessarily the one actually achieved.

**Table 2:** Evidence levels

Evidence level	Description
1a	A systematic review based on methodologically high-quality controlled randomized trials (RCTs)
1b	A sufficiently large, methodologically high-quality RCT
2a	A high-quality study without randomization, for example a cohort study
2b	A high-quality study of a different type of quasi-experimental study
3	A methodologically high-quality non-experimental study
4	Opinions and convictions of respected authorities (from clinical experience), expert commissions, descriptive studies

According to (Mehrholz 2010)

### Examination of the representation in various media

Subsequently, the presentation of the preparations on company websites, two selected online pharmacies, in specialist information and package leaflets was analyzed for the presence of information on the indication, ADRs, and interactions. Furthermore, it was recorded whether it was claimed that the preparation had an effect and whether this was also substantiated by studies (Table 3). In the case of company websites, all pages operated by the manufacturer of the preparation were included. This analysis was then extended for a detailed analysis of the compliance of company websites with the Therapeutic Product Advertising Act (Table 4).

**Table 3:** Presentation of homeopathic remedies on/in company websites, package leaflet, specialist information, OP1, and OP2 in percentage (%), as of 1 April 2023

	Presentation of indication	Presentation of ADR	Presentation of interactions	Claims about effectiveness	Presentation of studies
Company websites	87.50	25.00	37.50	100.00	56.25
Package leaflet	87.50	100.00	100.00	37.50	0.00
Specialist information	86.67	100.00	86.67	26.67	13.33
OP1	87.50	75.00	75.00	62.50	6.25
OP2	81.25	56.25	56.25	12.50	6.25

**Table 4:**
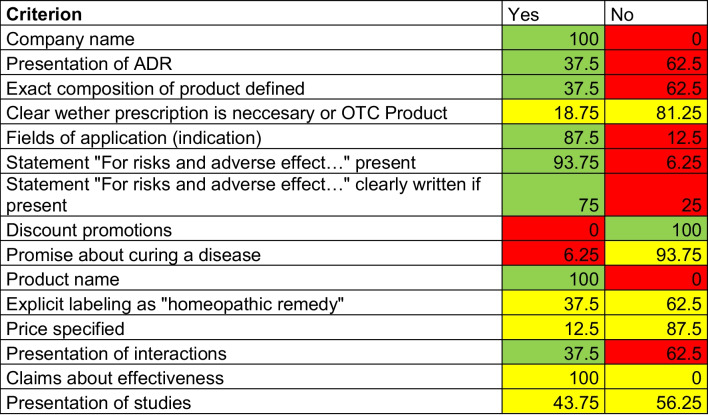
Compliance with the Therapeutic Products Advertising Act, presented in a table. Percentages are shown for each parameter. Green, compliance with legal regulations. Red, noncompliance with legal regulations. Yellow, ambiguous compliance with legal regulations, as of 11 December 2023

## Results

### Development of the DDD

**Fig. 1:**
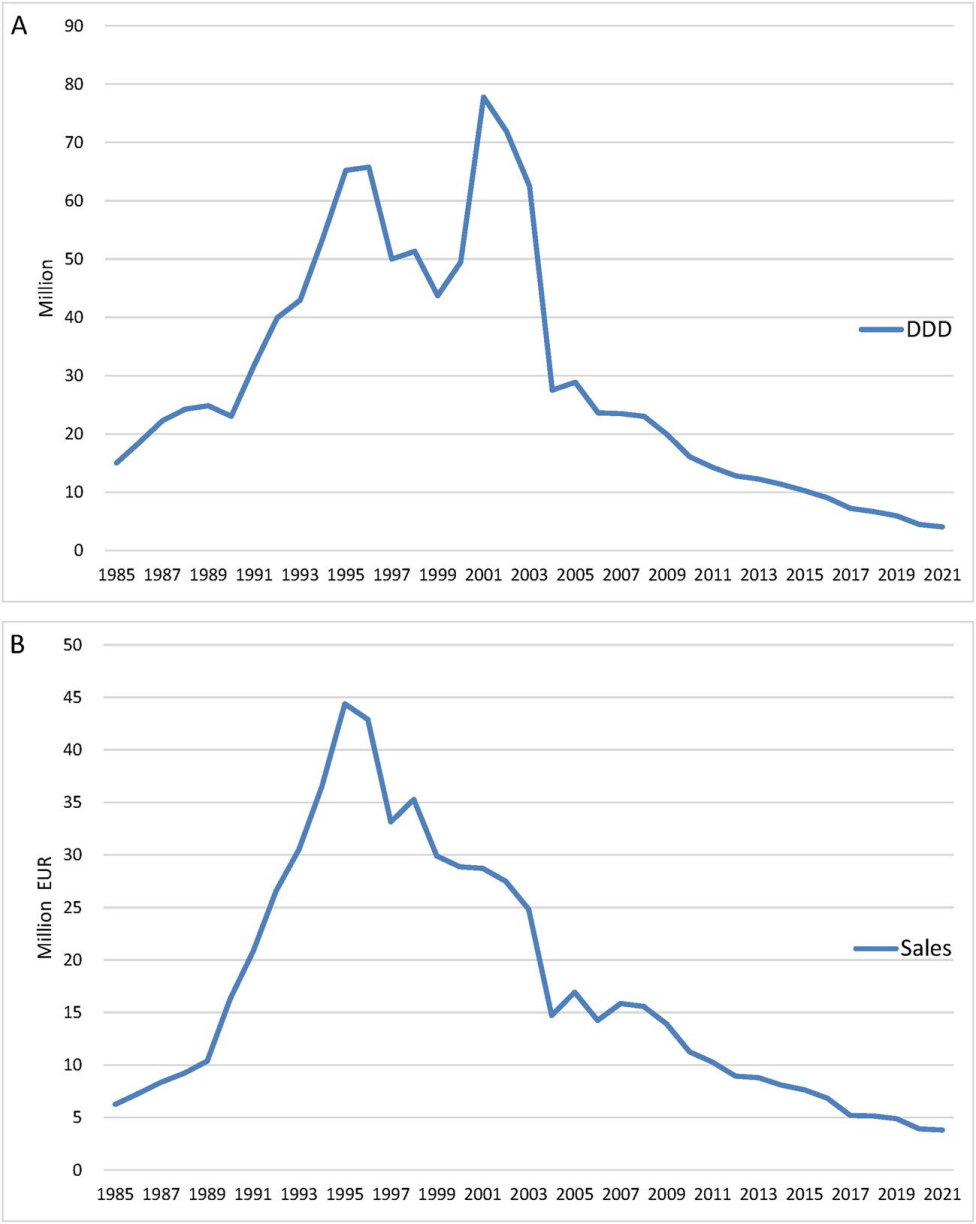
Development of DDD (**A**) and sales (**B**) from 1985 to 2021 in the SHI system for all analyzed preparations

**Fig. 2:**
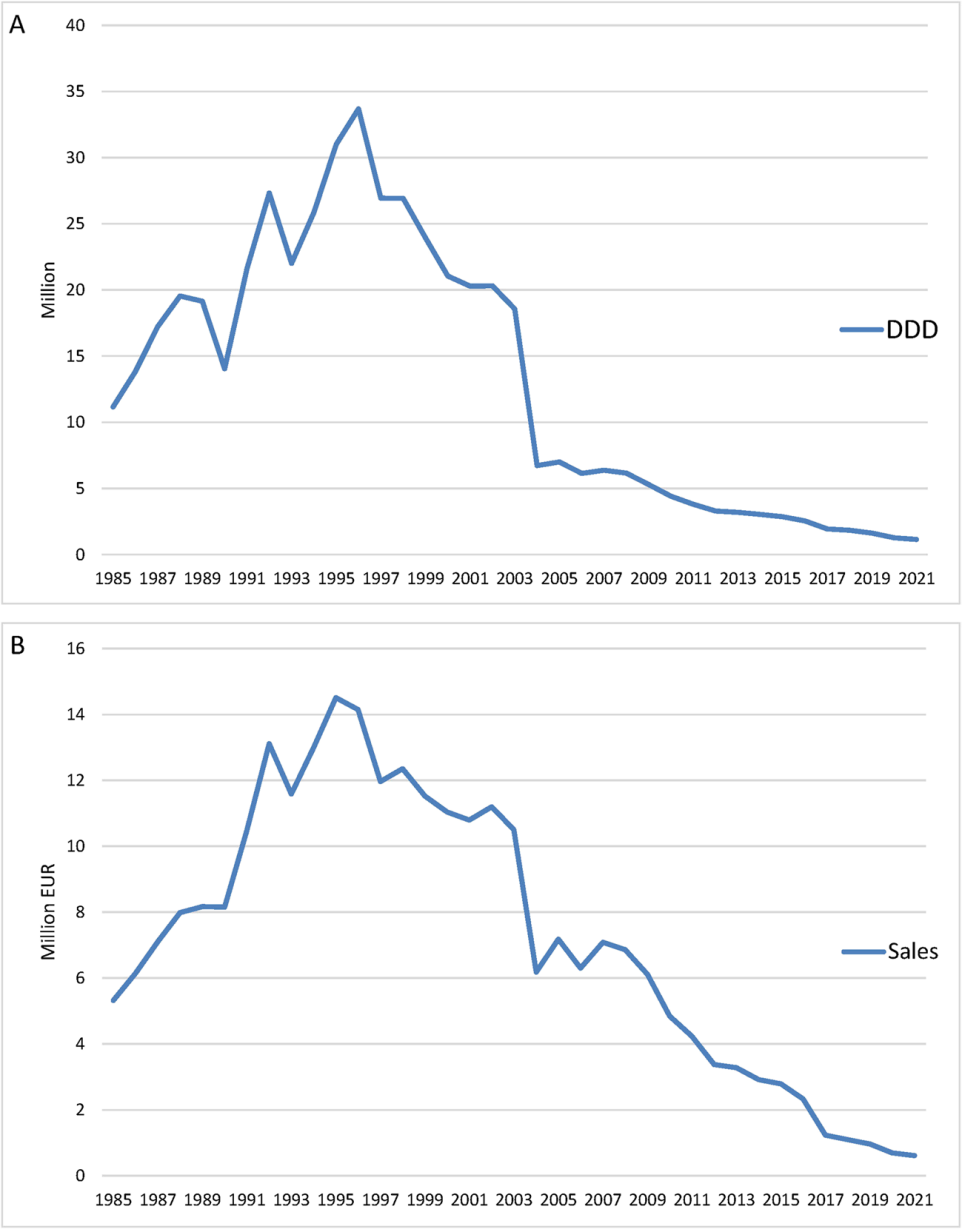
Development of DDD (**A**) and sales (**B**) from 1985 to 2021 in the SHI system for preparations that provide consistent data

**Fig. 3:**
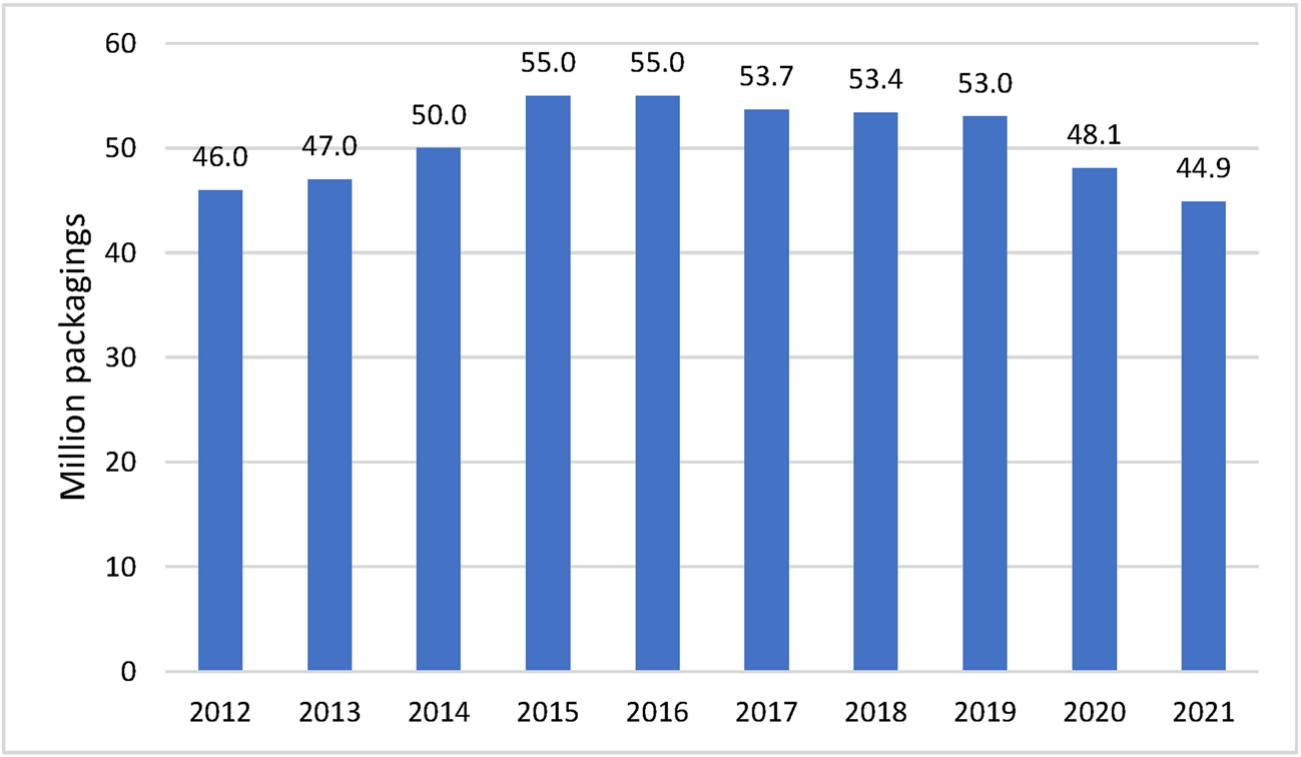
Overall sales development of homeopathic remedies in the pharmacy market in million packagings according to (Bundesverband der Pharmazeutischen Industrie e., V. (BPI) 2017) and (Bundesverband der Pharmazeutischen Industrie e., V. (BPI) 2023)

Figure 1 shows the overall DDD and sales of homeopathic remedies in the SHI system in Germany over time. Not every preparation is represented in every year, which is why the DDD and therefore also the total costs do not consistently represent the same pool of preparations. The reason for this lies in the WIdO data, which in some cases was unable to provide consistent data. HR1, which is heavily prescribed, has a significant influence on the curve. In 1995, the accumulated sales of the analyzed preparations reached its maximum at € 44,379,600. The maximum DDD was 77,771,100 in 2001, mainly due to the sharp increase in the prescription of HR1. A similar picture emerged when looking at the homeopathic remedies that provided data consistently from 1985 to 2021 (HR2 Ointment, HR5, HR6, HR16, HR12, HR14, HR13, HR2) (Fig. 2). Starting 2004 sales and DDD have decreased significantly. In 2021, the most recent sales were €3,809,000 while the DDD was 4,047,400. Figure 3 shows that the overall sales of homeopathic medicines remained fairly stable between 2012-2021.

### DDD costs

**Fig. 4:**
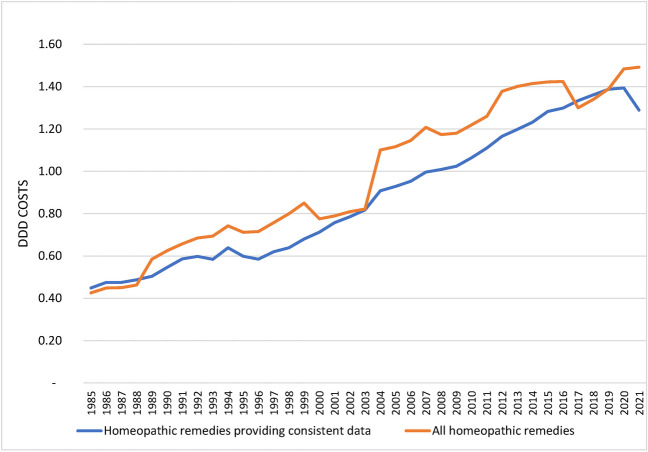
Development of DDD costs for all homeopathic medicinal products and for homeopathic medicinal products providing consistent data in the SHI system

**Fig. 5:**
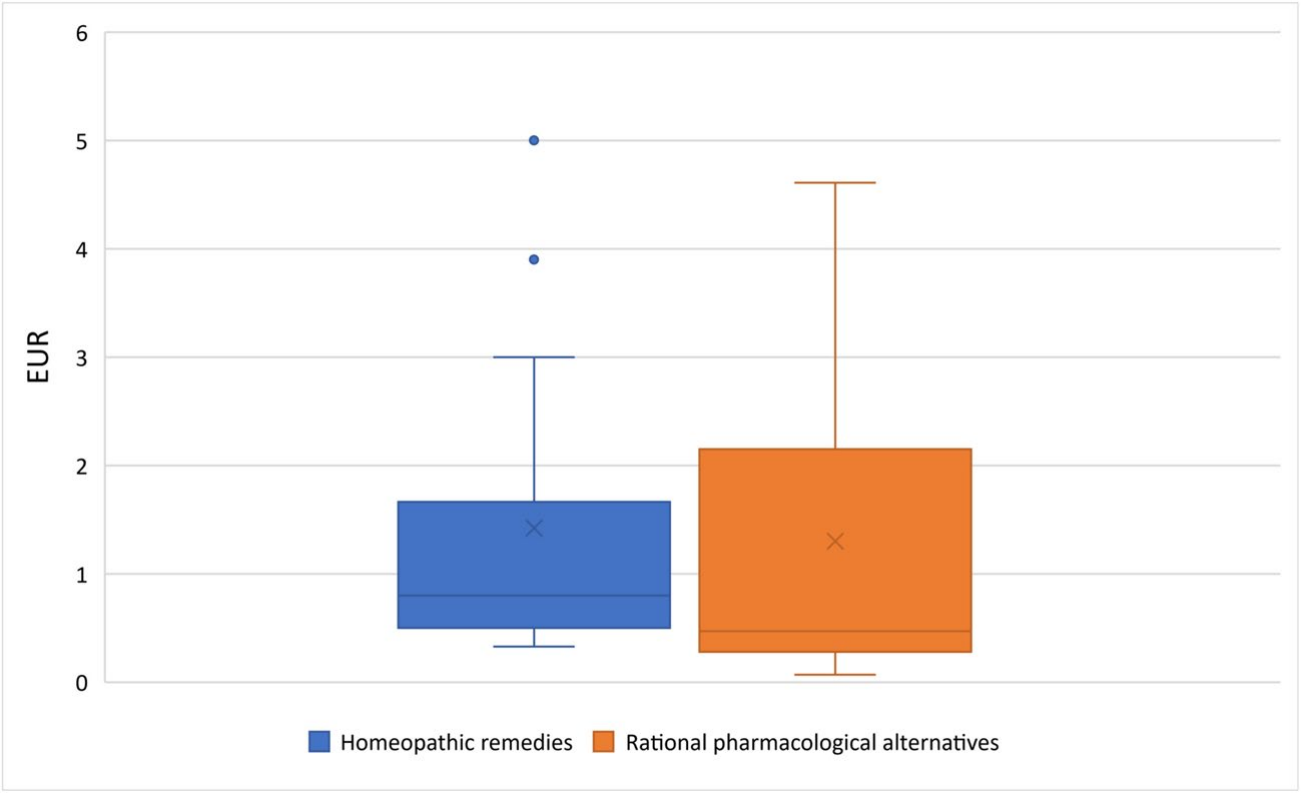
DDD costs in 2021 of homeopathics/rational pharmacological alternative

**Fig. 6:**
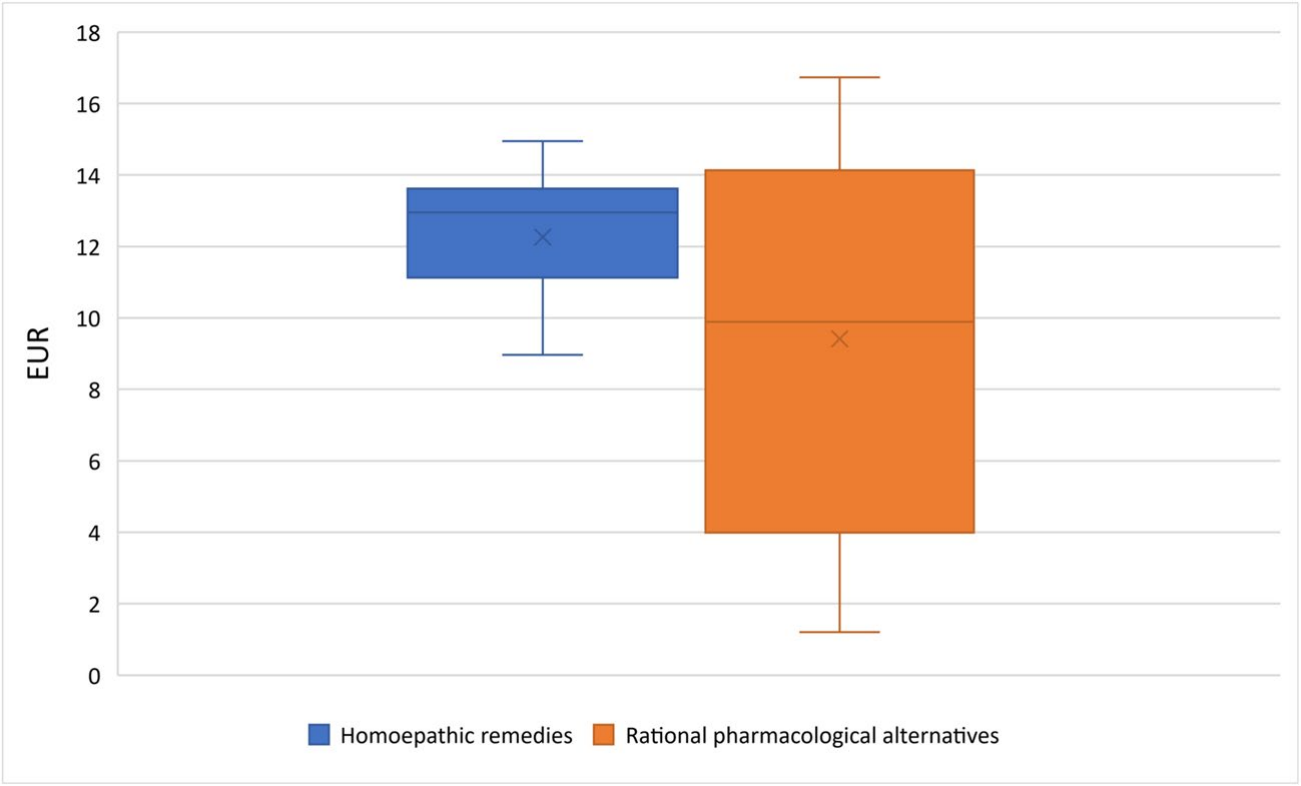
Pharmacy retail price of homeopathic remedies/rational pharmacological alternative for the smallest packaging in each case, as of 14 April 2023

As shown in Fig. 4, the average DDD costs for homeopathic remedies have risen from €0.43 in 1985 to €1.49 in 2021. The analysis of the homeopathic remedies providing consistent data also confirms this trend, even if there are slight deviations due to the data gaps in the graph of all homeopathic remedies analyzed. As can be seen in Fig. 5, the average DDD costs of the homeopathic medicines analyzed here in 2021 is €1.42, around 14% higher than that of the rational pharmacological alternatives at €1.30 while the average pharmacy price (as of 1 April 2023) of the smallest pack of homeopathic remedies is approx. 30% higher than that of rational pharmacological alternatives (Fig. 6). It should be noted here that we did not have any data on the DDD costs of HR15 for 2021, which is why the DDD costs for HR15 and its rational pharmacological alternative from 2019 were analyzed here as a proxy. Since the data of the WIdO could not provide the DDD costs for HR9 since 2011, this drug was not included in the analysis of the DDD costs of sales as in Fig. 6.

### Presentation of homeopathic remedies

Table 3 shows the presentation of the homeopathic remedies on company websites, two online pharmacies, in package leaflets and in specialist information. Company websites always stated that the preparation was effective and in 56.25% of cases this claim was also “substantiated” with studies (see section “Level of evidence in clinical studies”). In the other media, studies were cited much less frequently. For example, studies were only cited in 6.25% of cases in online pharmacies. It can also be seen that ADRs are the least frequently reported on company websites (25%) compared to other media. The same applies to interactions. The online pharmacies report ADRs and interactions much more frequently. At OP1 ADRs are stated in 75% of cases, at OP2 in 56.25% of cases. It should be noted in the presentation of the specialist information that there was no such information for one preparation (HR11). This preparation was therefore not included in the analysis of the specialist information. The reason why the indication was not always stated is due to the different authorization procedures (see section “Ways of market access for homeopathic medicinal products”) for homeopathic medicinal products. Legally speaking, registered homeopathic medicinal products do not have an indication (Bundesinstitut für Arzneimittel und Medizinprodukte (BfArM) 2023).

### Heilmittelwerbegesetz (Therapeutic Products Advertising Act)

In accordance with §4 HWG:

Any advertising for medicinal products within the meaning of § 2 para. 1 or para. 2 no. 1 of the Medicinal Products Act must contain the following information:

1. The name or company name and registered office of the pharmaceutical entrepreneur,
2. The name of the medicinal product,
3. The composition of the medicinal product pursuant to Sect. 11 para. 1 sentence 1 no. 6 letter d of the Medicinal Products Act,
4. The areas of application,
5. The contraindications,
6. The side effects,
7. Warnings, insofar as they are prescribed for the labelling of containers and outer packaging,
  7a In the case of medicinal products that may only be dispensed on medical, dental, or veterinary prescription, the words “Prescription only”,
8. The withdrawal period for medicinal products intended for use in food-producing animals.

### Analysis of manufacturer websites with respect to the Therapeutic Products Advertising Act

Table 4 shows how well the manufacturer complied with the Therapeutic Products Advertising Act on company websites. Therefore, we marked figures in compliance with this law in green and figures in non-compliance in red. Yellow figures represent parameters which are either not clear to be in accordance with the Therapeutic Products Advertising Act or not objectively determinable due to subjective influence. We observed non-compliance with the Therapeutic Products Drug Avertising Act in numerous categories, most strikingly ADRs, product composition, fields of application, interactions, and mandatory statements. In many important categories, it remained ambiguous whether the law was followed, specifically with respect to presentation of studies and effectiveness. Other formal criteria were also not clearly followed legally. Out of 30 table entries, just 9 received the color green, 11 the color yellow, and 10 the color red, visualizing the legal deficiencies at a glance.

### Level of evidence in clinical studies

**Fig. 7:**
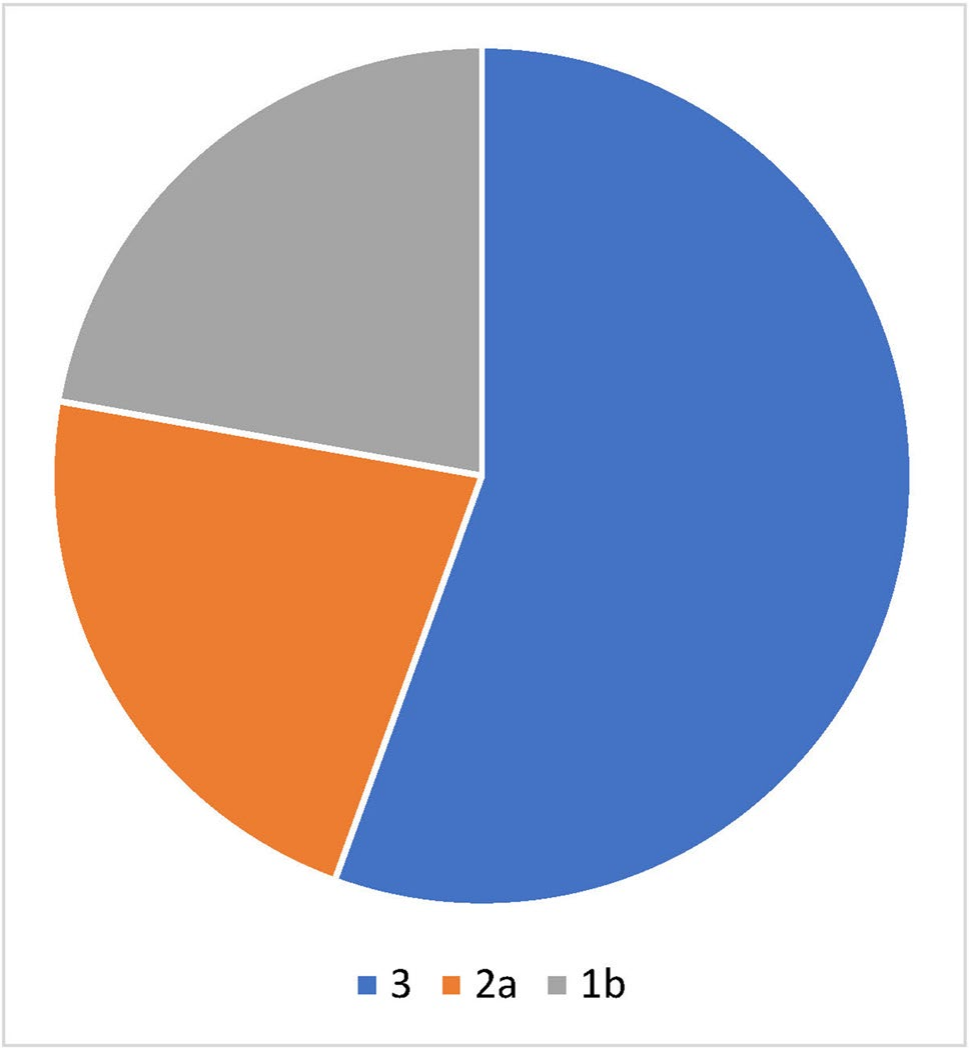
Aimed evidence level of clinical studies reported on company websites

**Fig. 8:**
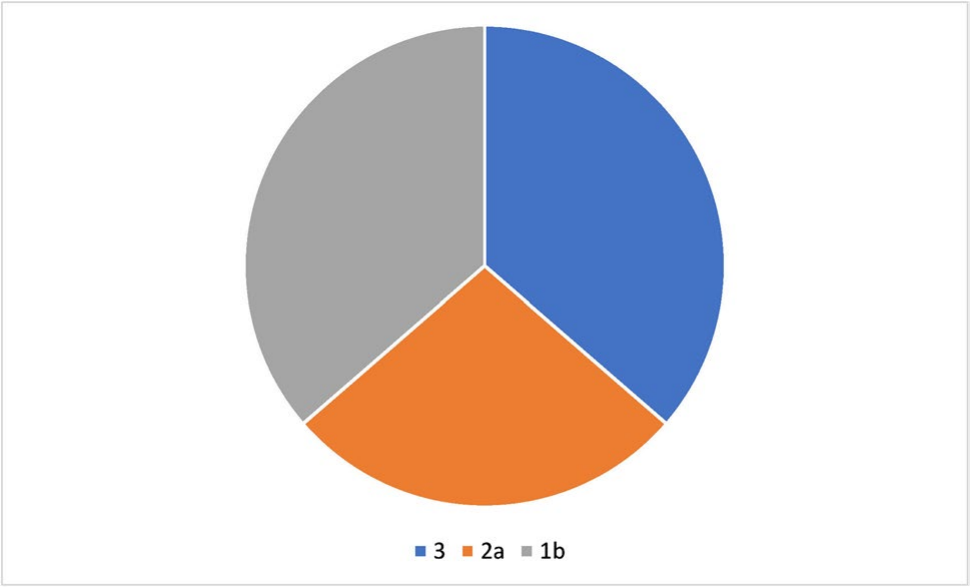
Aimed evidence level of clinical trials reported on PubMed

The level of evidence was determined for all studies specified by the manufacturer on the manufacturer’s official websites and for representative studies that could be found on PubMed for the respective preparation for the respective indication. For the registered homeopathic medicines, one indication was analyzed as an example. For HR2, this “indication” was musculoskeletal disorders and for HR7 intestinal colic/GI complaints. Studies that did not investigate these indications were not examined. In addition, only studies in which the forms of application were oral, topical, spray, or suppository, were considered. In addition, only studies that specifically examined the effect of the application of a single homeopathic remedy and not the effect of several homeopathic medicines together or in the context of another therapy were taken into account. In vitro studies were not included. In this way, nine studies were found for company websites (Fig. 7) and eleven for PubMed (Fig. 8). This revealed an uneven distribution on PubMed and company websites in favor of studies that aim for a higher level of evidence on PubMed. In other words, manufacturers mainly advertise studies with a low level of evidence. In most cases, these are observational studies (evidence class 3).

Among these clinical studies, we identified those for which access to the full texts was available. Thus, eleven studies could be found (Table 5). These studies were then analyzed regarding the study design (presence of randomization, placebo group, masking, ethical standards and primary outcome measure), possible bias (like sponsors/competing interests) and the journal in which the paper was published. Furthermore, we determined whether or not the study was registered and whether or not the conclusions of the paper were in favor of application of the homeopathic remedy or not. Cells with a green background represent factors that increases the scientific value of the paper. Red ones are factors that decrease the scientific value. Yellow represents factors which cannot be clearly assigned to one or another. Journals marked in green are those which are currently indexed for MEDLINE (https://www.ncbi.nlm.nih.gov/nlmcatalog/journals, as of 13 February 2024). Those marked in red are not indexed. It should be noted that access to studies could not always be provided since only two of six cited studies by the manufacturer of HR1 could be found. The majority of the analyzed studies revealed substantial deficiencies with respect to randomization, placebo group, making (blinding), and study type competing interest (sponsoring), resulting in some sort of bias in any given analyzed study. Although the majority of studies underwent peer review, the marked scientific deficiencies did not prevent the journals from publishing the studies. These findings show how important it is to perform post-publication peer review of peer-reviewed studies. Out of 121 table entries, 48 were coded green, 11 were coded yellow and 62 were coded red, highlighting the quality deficiencies of clinical studies at a glance.

**Table 5:**
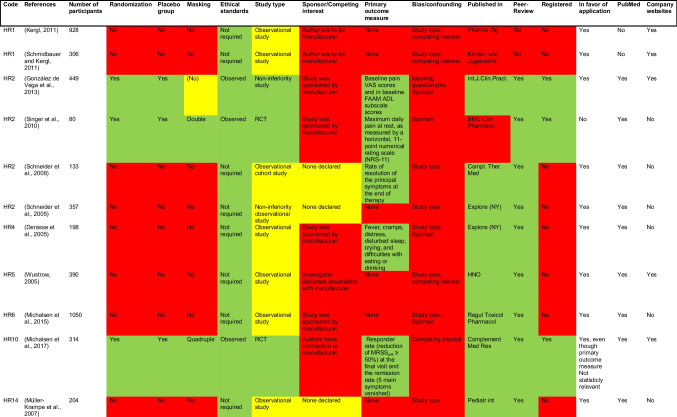
Detailed analysis of studies on company websites and PubMed, presented in a table. Green, factors that increases the scientific value of the paper. Red, factors that decrease the scientific value. Yellow, factors which cannot be clearly assigned to one or another, as of 19 December 2023

### Linguistic analysis of the names of the preparations

**Fig. 9:**
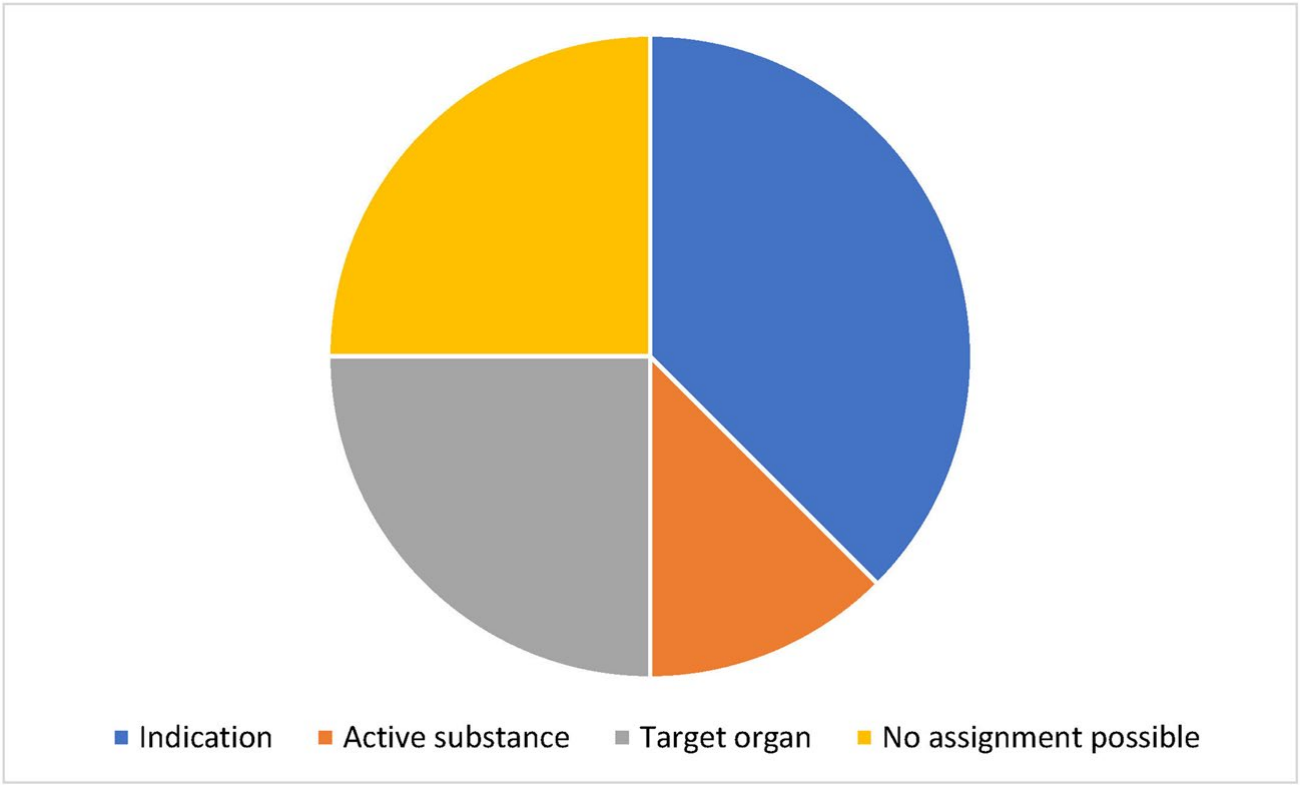
Linguistic analysis of the naming

The homeopathic remedies were categorized according to their names (Fig. 9). For 75% of the preparations, categorization according to indication, active substance, and target organ was possible. Here, too, the section “Ways of market access for homeopathic medicinal products” must be observed for the registered homeopathic remedies, as the naming can convey an indication to the user.

### Application and dosage forms

**Fig. 10:**
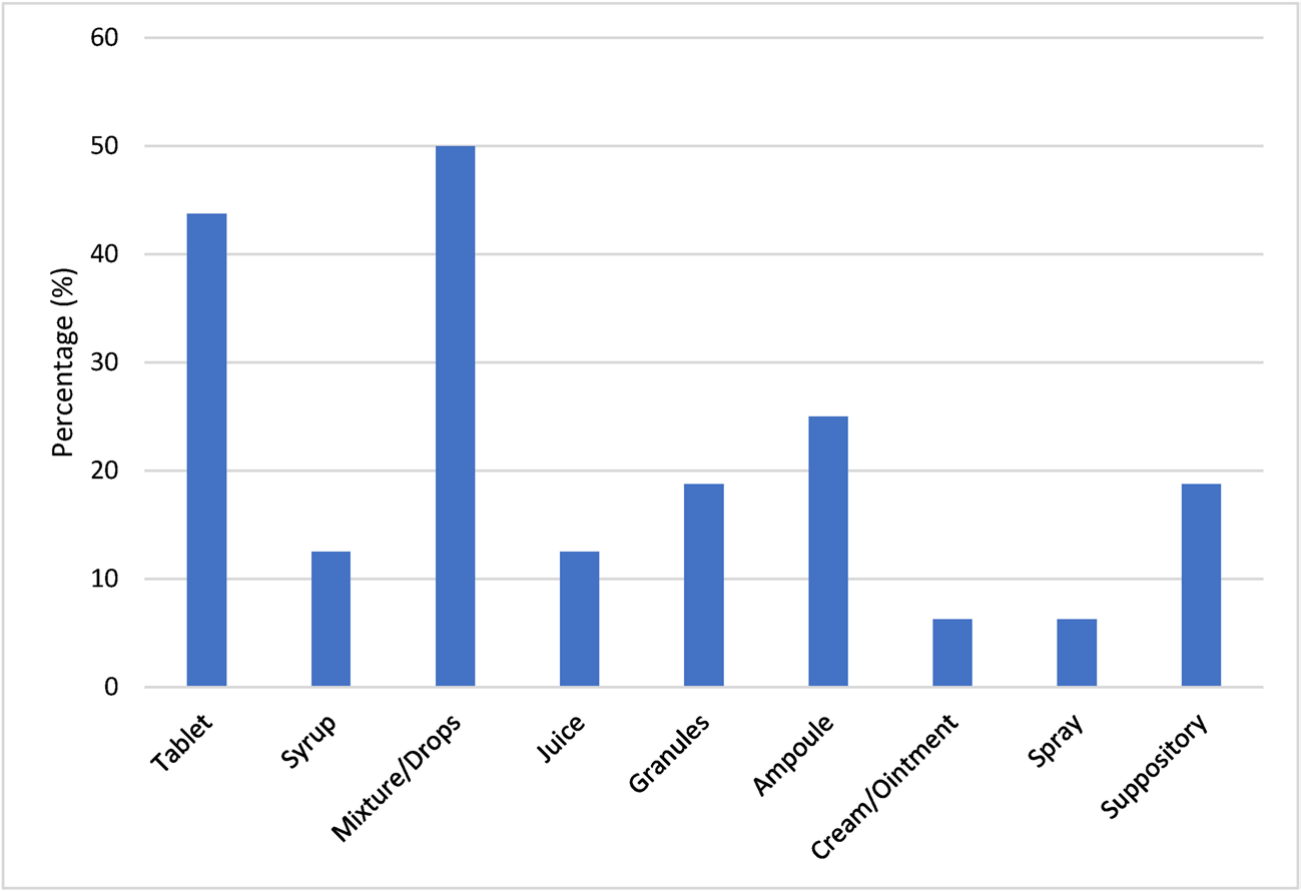
Application forms of homeopathic remedies. Percentages of all examined homeopathic remedies that are available in this application form

Figure 10 shows the application forms of the homeopathic remedies analyzed. The most common application form was the mixture/drops, closely followed by the tablet with 44%. Three of the 16 preparations are available in the form of granules. It is striking that 25% of the preparations are available in the form of ampoules, which are intended for intramuscular, intravenous and intra-articular injection. Parenteral drug applications are potentially dangerous in terms of causing allergic reactions and/or infections at the site of injection.

### Exemplary analysis of selected clinical studies

#### HR2

As a registered homeopathic medicinal product, HR2 has no specified indications. Nevertheless, the preparation is generally used for musculoskeletal disorders. In 2013, the manufacturer published a study to prove the non-inferiority of HR2 to diclofenac in reducing pain and improving ankle mobility in cases of ankle sprains. The study design was randomized, double-blind and controlled. A total of 449 study participants were divided into three treatment arms and treated with either HR2 Ointment, HR2 Gel, or diclofenac 1% topical. The results of the study showed non-inferiority of both HR2 ointment and gel to diclofenac (González de Vega et al. 2013).

On closer inspection, however, this study raises some questions. As HR2 ointment and diclofenac gel have different colors, and there was no double-blinding here. It is also possible that the 1% concentration of diclofenac was not high enough to have a therapeutic effect (Kerschner 2018). An additional placebo arm would have remedied this methodological problem.

#### HR1

HR1 is an authorized homeopathic medicine for the treatment of colds. The effect of this preparation was tested in an observational study with 1115 patients in 2015. The primary study objectives were assessment of the efficacy and tolerability of HR1 by the patient based on the evaluation of anonymized questionnaires.

The subjective perception of the improvement of individual cold symptoms and the effectiveness of the remedy were investigated by asking how satisfied the patient was with the effect (Gerke and Schäkermann 2015). As 90% of patients were satisfied or very satisfied with the effectiveness, the HR1 website (MEDICE Arzneimittel 2023) concluded that:

1. HR1 is able to achieve a fast and reliable reduction of all the typical cold symptoms.
2. This pharmacy-based observational study proves once again the efficacy and tolerability of HR1
3. HR1 activates the body`s self-healing powers in a holistic approach which is an advantage compared to chemically synthesized drugs which only suppress the symptoms.

Following a legal dispute between the Verbraucherzentrale NRW and the manufacturer of HR1, the Landesgericht Dortmund ruled in 2022 that this constituted misleading advertising in accordance with § 3 S.2 Nr. 2a HWG (Sucker-Sket 2023).

### In vitro studies

Many manufacturers also use in vitro studies to convince potential buyers of their product (e.g., HR3). The Benviste affair shows how dangerous it is when in vitro studies are used to prove the alleged effect of homeopathic remedies (Maddox et al. 1988). Benviste examined high potencies beyond the Avogrado constant (Davenas et al. 1988) whereas only low potencies were investigated in this study. Nevertheless, no effect on humans can be easily deduced from the results of these in-vitro studies.

## Discussion

### Development of DDD and sales

The developments of the DDD and thus also the resulting parameters are only meaningful until 2004, as homeopathy was removed from the SHI benefits catalogue as of 2004 because of the SHI Modernization Act. To this day, homeopathic remedies are only paid for as a voluntary statutory benefit. As these are not covered by the usual “statutory health insurance prescription,” these statutory benefits are not recorded by the WIdO. Even when we enquired with four major health insurance companies in Germany, they were unable to provide us with any data in this regard. Although it is generally no longer possible to obtain homeopathic remedies via a “health insurance prescription,” it is still possible for children under the age of 12 and adolescents with developmental disorders up to the age of 18 to obtain homeopathic remedies this way. This inevitably means that the selected preparations, which are those that have appeared in the AVR since 2005, are primarily used for pediatric indications.

Even if the data from 2004 onwards no longer reflects the total expenditures on homeopathic medicines covered by the SHI, it can still be seen that the DDD and sales reimbursed via “health insurance prescription” fell after 2004, while both the pharmacy retail price and the DDD costs have increased significantly. Based on data from the Bundesverband der Pharmazeutischen Industrie (Bundesverband der Pharmazeutischen Industrie e., V. (BPI) 2023) (see Fig. 3), it can also be seen that total sales of homeopathic medicines have remained fairly constant at a high level from 2012-2021. It can therefore be assumed that there has been a shift from prescriptions via the “health insurance prescription” to the “private prescription” or OTC purchase. 

### Ways of market access for homeopathic medicinal products

In Germany, there are two legal ways through which homeopathic medicinal products can enter the market: authorization (Zulassung) and registration (Registrierung).

### Authorization

Homeopathic remedies labeled with a specific indication can only be sold if they receive authorization by the BfArM. The efficacy of homeopathic remedies is not proven via a clinical efficacy test, but via the BfArM’s *“Beurteilungskriterien für homöopathisches Erkenntnismaterial” (Assessment criteria for homeopathic knowledge material). An* expert judgement (consensus conference of homeopaths) and long-term use (at least since 1978) are sufficient for the approval of a medicine for minor illnesses (Bundesinstitut für Arzneimittel und Medizinprodukte (BfArM) 2023). From a legal point of view, the manufacturer has then fulfilled its obligation to provide proof of efficacy, even though this could hardly be considered proof of efficacy according to scientific standards.

### Registration

If homeopathic medicinal products are to be placed on the market without a specific indication, they can be registered. However, this option is only available for those remedies which are used orally or externally and the homeopathic starting material is diluted by a factor of at least 1:10,000 (potency D4/C2, see § 38 AMG and § 39 AMG).

Since registered homeopathic medicinal products are placed on the market without specific indication, the legislator has not installed any obligation of the manufacturers to prove the efficacy of the product (Bundesinstitut für Arzneimittel und Medizinprodukte (BfArM) 2023).

### Clinical study quality in a legal context

The studies that the manufacturers of homeopathic remedies state on their websites are usually classified as evidence class 3 (mostly observational studies) and are not sufficient to prove the clinical efficacy of a preparation beyond doubt. In addition, there are often methodological flaws in these studies, as discussed in the section “Exemplary analysis of selected clinical studies.” The uneven distribution of evidence levels between PubMed and company websites suggests that manufacturers tend to refer to studies with a lower level of evidence because these are more often in their favor. These results would also be congruent with the publication bias known in homeopathy (Gartlehner et al. 2022). Another explanation would be that methodologically high-quality studies are more expensive and that manufacturers refrain from conducting them for cost reasons because they may already assume that the result would not be in favor of the homeopathic remedy.

Homeopathy advocates often claim that the efficacy of homeopathic remedies cannot be clinically proven, as the indication according to classical homeopathic teaching is too individual to determine a statistically significant difference to the placebo in large-scale studies (Lifeline 2022). However, since the investigated preparations are merely homeopathic complex preparations, for which even the manufacturer often states a broad indication such as cold symptoms, this argument loses its significance. Nevertheless, none of the preparations investigated provided adequate proof of efficacy, as would be necessary, for example, for the authorization of a “normal” drug. Instead, studies with little significance for the actual effect are used to convey to the patient that proof of efficacy has actually been provided.

Even if, according to the *“Beurteilungskriterien für homöopathisches Erkenntnismaterial” (Assessment criteria for homeopathic knowledge material)* (Bundesinstitut für Arzneimittel und Medizinprodukte (BfArM) 2023) points for authorization can be collected with, e.g., observational studies, the main motivation of homeopathic manufacturers to publish studies on efficacy is probably marketing, because so far, no homeopathic medicinal product has been authorized by the BfArM for which the applicant has referred to a study (Bundesinstitut für Arzneimittel und Medizinprodukte (BfArM) 2023).

The currently valid legal position therefore allows the manufacturers of homeopathic remedies to repeatedly advertise their studies, which have never been submitted to the BfArM, as proof of efficacy.

On the contrary, registered homeopathic remedies, such as HR2 or HR7, do not have to provide proof of efficacy for their registration at all, but are also not allowed to state an indication. In this context, the advertising of HR2, for example, is more than questionable, as it clearly targets musculoskeletal complaints, even if the indication is not directly stated because homeopathic medicinal products that are registered or exempt from registration under the German Medicinal Product Act may not be advertised with indications for use (see §5 HWG).

Nevertheless, authorized homeopathic remedies in Germany are certified as effective via the special authorization procedure, although this regulation in particular has no scientific basis and should be critically reconsidered. It can be stated that homeopathic medicinal products are privileged in many respects in their ability to gain market access.

### Inconsistency of information in advertisements

This special authorization procedure is particularly problematic in the case of the investigated complex preparations with relatively low potencies, as they have the potential to be toxicologically effective. This is why the INH (Informationsnetzwerk Homöopathie) with regard to consumer safety advises to subject homeopathic complex remedies with low-potency herbal substances to scientific evaluation since the potential of interaction of plant extracts with each other or with other pharmaceuticals may be clinically relevant (INH 2020). In this context, the inconsistency of information in various documentation and various online pharmacies must be viewed critically, as, for example, only 37.5% of interactions are listed on company websites (see Table 3).

Moreover, adverse effects of homeopathy are frequently reported in homeopathic trials (Stub et al. 2012, 2016, 2022) and can be caused by allergic reactions or ingestion of toxic substances. For example, remedies with heavy metals which are often used in homeopathy can cause toxic reactions (Posadzki et al. 2012). The spectrum of ADR caused by homeopathic remedies ranges from relatively harmless symptoms like abdominal pain and flatulence to severe allergic reactions, bladder cancer, or acute pancreatitis (Posadzki et al. 2012).

Even though ADR are more frequently reported in conventional medicine (Stub et al. 2022), one should still consider that not only the absolute risk of the treatment but also the risk–benefit balance is important to evaluate the use of this particular treatment (Posadzki et al. 2012). Thus, a minimal or no effect of a medical treatment can hardly be justified.

94.7% of ADR of homeopathic remedies are caused by those with a potency lower than C12/D24 (Posadzki et al. 2012). The highest dilution investigated in this paper, however, is C8 (HR11), which means that there is a risk of ADR, particularly for the homeopathic remedies investigated in this paper. Consequently, Table 4 is worrying as certain requirements of the Therapeutic Product Advertising Act such as the presentation of the ADR, interactions, or the exact composition of the defined medicinal product are not adequately met in most cases. However, the mentioned violations of the Therapeutic Product Advertising Act are not unique to homeopathy, but occur more broadly, as was recently shown for drug advertisements in the popular German health magazine “Apotheken Umschau” (Keuper and Seifert 2023).   

### Price comparison

Finally, the price comparison shows that the homeopathic remedies are more expensive than their rational alternatives, which is astonishing, especially if we assume that these remedies do not work better than a placebo.

### Limitations

The data provided by the WIdO do not provide consistent information for every preparation, which means that there can be strong fluctuations in the course of the DDD, for example, if a preparation is listed in the AVR for the first time in a given year. Nevertheless, the data provide a good overview of the development of the homeopathic remedies listed in the AVR from 2005 onwards. The WIdO data only include medicines prescribed by registered doctors in outpatient care and dispensed via public pharmacies at the expense of the SHI. In addition, only dispensing via the SHI prescription is recorded, not via private prescriptions.

As from 2004 onwards, homeopathic remedies can only be prescribed for children up to the age of 12 or adolescents with developmental disorders up to the age of 18 via the SHI prescription, the majority of the preparations examined are used for pediatric indications. Furthermore, the figures from 2004 onwards no longer reflect the actual proportion paid for by the SHI, but only that for the pediatric indications in question.

The majority of clinical studies analyzed in this paper (Table 5) reported a positive effect of homeopathic medicines and favored application of such drugs. It is well known that there is a strong bias towards publication of positive data in the medical literature (Easterbrook et al. 1991), and we have no information how many negative studies on homeopathic medicines were performed and never published.

## Conclusions

In conclusion we have shown that homeopathic medicines in Germany are still important to the drug market, with a clear shift from SHI prescriptions to non-SHI prescriptions and OTC use for which we have only very limited data. The DDD costs for homeopathic medicines have substantially increased over the years, and homeopathic medicines are often more expensive than rational (and pharmacologically effective) alternatives. We noted multiple deficiencies in the presentation of homeopathic medicines in various media and substantial non-compliance with the Therapeutic Product Advertising Act. Suggestive product naming is a problem, too. Moreover, some parenteral homeopathic medicines are potentially dangerous. Lastly and most importantly, the overall quality of clinical studies dealing with homeopathic medicines is poor. In conclusion, our study provides pharmacoeconomic, legal, and pharmacological arguments for discussion on the future of homeopathic medicine at the expense of the SHI system in Germany. Our data support the initiative of the German Federal Health Minister, Prof. Dr. Karl Lauterbach, that for scientific, legal and pharmacoeconomic reasons, reimbursement of homeopathic remedies should be excluded from the SHI system.

## Data Availability

All source data for this study are available from the authors upon reasonable request.

## Abbreviations

SHI: Statutory health insurance
BfArM: Bundesinstitut für Arzneimittel und Medizinprodukte (Federal Institute for Drugs and Medical Devices)
ADR: Adverse drug reaction
DDD: Defined daily dose
AVR: Arzneiverordnungsreport (Drug prescription report)
WIdO: Wissenschaftliches Institut der Ortskrankenkassen (AOK) (Scientific Institute of the General Local Health Insurance Fund, AOK)
HWG: Heilmittelwerbegesetz (Therapeutic Products Advertising Act)
HR: Homeopathic remedy
OP: Online pharmacy
OTC: Over-the-counter
